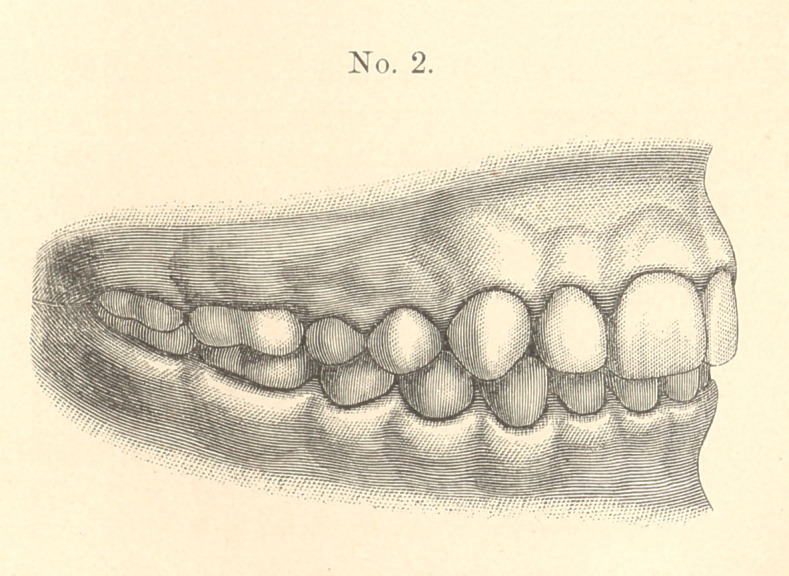# Description of a Case of Regulating

**Published:** 1892-06

**Authors:** H. F. Hamilton

**Affiliations:** Boston


### DESCRIPTION OF A CASE OF REGULATING.[1]

BY DR. H. F. HAMILTON, BOSTON.

THE case I present to-night is a common one, but the treatment is, I think, new. The patient, a boy of thirteen, had the condition of teeth shown in model marked No. 1, taken December 1, 1[illegible]

[1] Read before the American Academy of Dental Science, January 6, 189[illegible]

No. 1.

No. 2.

The lower jaw was receding, and the upper front teeth were so prominent as to prevent the easy closing of the lips, giving the face the weak expression characteristic of these cases.

Pulling in the upper front teeth would only improve the facial appearance slightly, so I determined to try throwing the whole lower jaw forward the diameter of a bicuspid. This I accomplished easily, so that in four months the condition was as shown in model No. 2.

You will understand what was done by noticing the articulation. In No. 1 the superior second bicuspid strikes in front of the inferior second bicuspid. In No. 2, you will notice, it strikes behind. The same change is, of course, shown with all the other bicuspids and molars.

It was finished nearly six years ago, and I have watched it carefully before reporting. The teeth have not changed, and the wonderful improvement then shown in the boy's appearance has increased, the face being now strong rather than weak.

The method used was simply a rubber plate fitting the roof of the mouth and over the bicuspids and first molars, where it was made thick, and with depressions to receive the cusps of the lower teeth. But these depressions, instead of being directly over the cusps, were slightly in front, so as to throw the lower jaw forward when closed, by the action, as it were, of a series of inclined planes.

A plate was made also for the lower jaw on the same plan, and worn alternately to keep the teeth in place, and to avoid the injury likely to come from long and continuous wearing of plates.

At the end of four months the second molars, not being covered by the plates, had grown together so as to articulate when the plate was in place, and the result of this change was that the patient could not close the teeth in the original manner. The changed position was the only comfortable one.

The plates were worn but little after this, nor were stay plates of any kind necessary.

This advance of the lower jaw comes, I think, from preventing the condyle going to its place; but there is a change going on at the angle of the jaw which will ultimately allow a perfect articulation.

This explanation is theoretical, and I should be glad of light on the subject.

## Figures and Tables

**No. 1. f1:**
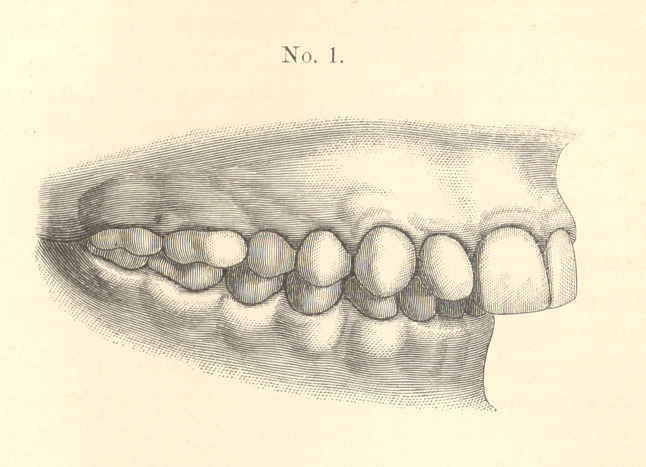


**No. 2. f2:**